# Reframing trauma-informed care for refugee and (im)migrant health systems: from interpersonal practice to structurally embedded care

**DOI:** 10.3389/frhs.2026.1882791

**Published:** 2026-07-10

**Authors:** Hyojin Im, Sophia Banu, Aisha Koroma, Sarmaya Mustafayeva

**Affiliations:** 1 School of Social Work, Virginia Commonwealth University, Richmond, VA, United States; 2 Menninger Department of Psychiatry and Behavioral Sciences, Baylor College of Medicine, Houston, Houston, TX, United States; 3 HIAS, Silver Spring, MD, United States

**Keywords:** administrative burden, health equity, mental health services, migrants and immigrants, refugees and asylum seekers, refugee resettlement, trauma-informed care (TIC)

## 1 Introduction: TIC in refugee & im(migrant) health systems

Refugee and (im)migrant health services are increasingly incorporating elements of trauma-informed care (TIC), yet remain unevenly equipped to fully address the complexity of trauma in contexts of forced migration. Trauma-informed care (TIC) refers to an organizational approach that recognizes the pervasive effects of trauma and integrates this knowledge into policies, procedures, and everyday practices that promote safety, trust, collaboration, empowerment, and cultural responsiveness (1). We use trauma to refer not only to the effects of discrete events, but also to the lasting psychological, embodied, and relational consequences of harmful circumstances that overwhelm safety, control, dignity, or belonging. In refugee and (im)migrant contexts, such circumstances may include exposure to violence or persecution before and during migration, as well as post-migration stressors such as legal precarity, family separation, administrative instability, exclusion, and barriers to care (2). Although refugees, asylum seekers, immigrants, and migrants differ in legal status, migration pathways, trauma exposures, and service eligibility, they are often served within overlapping health and social service systems. Across these settings, shared structural mechanisms, including legal precarity, administrative burden, fragmented services, and exclusionary policy environments, shape trauma and care in ways that vary by status and context. Over the past decade, many institutions have adopted trauma-informed approaches through staff training, trauma screening protocols, and efforts to improve provider awareness of trauma histories (3). TIC has helped name these practices, but institutions have not consistently resourced, protected, or systematized them, and substantial gaps remain in implementation and capacity (3, 4). While TIC has improved provider awareness and relational practices, its growing institutional adoption risks obscuring the structural and political conditions that continue to re-traumatize refugees and (im)migrant populations (5, 6). TIC has largely been practiced within clinical and interpersonal frameworks, limiting its capacity to address the structural conditions that shape trauma in refugee contexts. In health systems, trauma-informed practice is operationalized as a set of provider competencies, communication strategies, or screening tools designed to reduce re-traumatization and improve patient engagement (1, 7). For refugees and asylum seekers, however, trauma is rarely confined to the past, and experiences of forced migration are frequently followed by prolonged asylum procedures, temporary or uncertain legal status, documentation instability, and barriers to employment, housing, transportation and health care. These conditions create persistent uncertainty and insecurity long after arrival in receiving countries (8). As a result, trauma in refugee and (im)migrant contexts is not only a past exposure or individual clinical history; it can also be sustained when receiving-country systems repeatedly expose people to uncertainty, loss of control, conditional access to resources, and prolonged waiting. Health systems serving displaced populations operate at the intersection of clinical care and multiple bureaucratic regimes, including immigration adjudication, housing systems, and public benefits programs. Administrative delays, repeated eligibility verification, documentation requirements, and health coverage transitions can therefore sustain chronic stress and undermine recovery (9). In this context, TIC cannot be meaningfully confined to interpersonal clinical practice. The central challenge is not simply a deficit of compassion or commitment among providers, but a structural misalignment between clinical care models and the policy environments that reproduce instability, bureaucratic friction, and administrative lag, generating chronic uncertainty.

This paper argues that TIC, as currently operationalized, remains insufficient for refugee-serving systems because it is overly confined to interpersonal and clinical domains. We propose a reconceptualization of TIC as a structurally embedded, system-level approach that integrates administrative, legal, and policy conditions as core determinants of trauma and healing. Although these structural mechanisms are relevant across many Western resettlement contexts, this paper draws primarily on the United States health and social service system. U.S.-based examples are used to illustrate broader patterns of administrative burden and structural misalignment, while recognizing that these processes vary across welfare regimes, migration policies, language access systems, and institutional contexts.

## 2 Structural misalignment: trauma-informed care in trauma-producing systems

For refugees and asylum seekers, trauma cannot be understood as an individual condition alone. It is also shaped by the legal, administrative, and policy environments through which displaced people are required to seek safety, care, and belonging. Legal precarity, bureaucratic uncertainty, and exclusionary policy regimes can generate cumulative psychological harm by repeatedly constraining access to stability, resources, and social participation (10). For example, temporary or unresolved immigration status constrains access to employment, housing, and public benefits while prolonging uncertainty regarding long-term stability. Prolonged asylum processes have been consistently associated with elevated rates of depression, anxiety, and posttraumatic stress symptoms (11, 12). These harms are not merely downstream effects of displacement. They are ongoing conditions through which distress is organized and sustained.

Administrative burden is one key mechanism through which institutions produce distress. Refugees and asylum seekers often navigate repeated documentation requirements, eligibility verification, delays, and discretionary gatekeeping across health care, immigration, housing, and public benefit systems (13, 14). Medicaid interruptions after status transitions, disability benefit denials despite documented impairment, repeated requests for records already submitted, and prolonged case backlogs can suspend access to treatment, housing, or family reunification (15, 16). These processes impose cumulative cognitive and emotional demands under conditions of uncertainty and limited control. In this sense, administrative systems do not merely mediate access to care. They actively participate in the production of distress, which creates a fundamental tension. TIC is implemented within systems that simultaneously generate the instability it seeks to mitigate. While providers are tasked with fostering safety, trust, and continuity, institutional processes routinely introduce disruption, unpredictability, and exclusion. The result is not simply a gap in implementation, but a structural misalignment between the goals of care and the conditions under which care is delivered. The problem is not merely inadequate implementation of TIC, but the assumption that trauma can be addressed primarily through interpersonal intervention while structural conditions remain unchanged. This conceptual narrowing has organizational consequences. When refugee trauma is framed primarily through individual psychiatric categories, particularly PTSD, organizations may focus on screening, referral, and symptom management while treating legal, administrative, and socioeconomic conditions as external context.

This disconnect becomes visible at the level of organizational implementation. Although trauma-informed principles are widely endorsed, their translation into practice remains uneven and often superficial. Many TIC models remain anchored in Western psychiatric frameworks, particularly PTSD as the dominant lens for understanding refugee distress (6, 17). While clinically useful in some contexts, this framing can narrow attention away from structural determinants such as legal precarity, family separation, racism, and social exclusion (18). Standardized trauma screening tools may inadvertently pathologize adaptive responses to chronic uncertainty, producing forms of diagnostic reductionism that are misaligned with the lived realities of displaced populations (19). In some cases, disclosure of trauma may carry legal or social risks, particularly for individuals navigating asylum processes or child welfare systems, thereby complicating assumptions about the benefits of routine screening (20).

At the same time, organizational structures often fail to support the relational practices that TIC requires. Training is frequently optional or delivered without protected time, and reflective supervision is encouraged but not systematically institutionalized (21, 22). Performance metrics tend to prioritize service volume, documentation, and compliance rather than relational continuity or staff well-being. In response, practitioners often compensate through discretionary effort, extending work hours or providing additional support beyond formal role expectations. While such efforts sustain care in the short term, they also contribute to workforce burnout and turnover over time. These limitations are not incidental. They reflect systems organized around service volume, documentation, compliance, and short-term outputs, while the relational work central to TIC remains difficult to quantify and rarely reimbursed. Providers are expected to deliver TIC within administrative and funding structures that often make trauma-informed practice difficult to sustain. This produces a persistent paradox: TIC is promoted within systems that simultaneously generate conditions of chronic uncertainty for both clients and providers. Over time, this dynamic contributes not only to variability in service quality but also to workforce instability, moral distress, and moral injury, as practitioners confront the limits of what can be achieved within structurally constrained environments (23–25).

## 3 The relational infrastructure of trauma-informed care

This structural misalignment does not mean that refugee-serving systems lack trauma-informed practice. On the contrary, many already contain a relational infrastructure of TIC: informal networks of trust, cultural knowledge, discretionary labor, and everyday practices through which frontline workers create safety, continuity, and meaning in service encounters (26, 27). Unlike protocols, screening tools, or training modules, this infrastructure is often informal, embodied in frontline work, and sustained through discretionary labor. Across refugee-serving organizations, case managers, interpreters, community health workers, peer navigators, resettlement staff, and other frontline workers routinely provide emotional containment, pacing, trust-building, crisis buffering, and institutional translation. They may adjust intake procedures when clients are overwhelmed, sequence documentation around family crises, or explain bureaucratic processes in ways that reduce fear and confusion. Such practices enact core trauma-informed principles, particularly safety, trustworthiness, collaboration, and empowerment.

Staff with lived experience of migration or displacement are central to this infrastructure. In many refugee-serving organizations, they function as cultural brokers, community connectors, interpreters of institutional expectations, and trusted intermediaries between clients and formal systems (28). Their credibility within refugee communities can facilitate trust and engagement in ways that formal procedures alone rarely achieve. These relational dynamics are not merely interpersonal qualities but mechanisms through which system-level outcomes are produced. Trust, engagement, care continuity, and service navigation emerge as relational achievements rooted in shared experience, cultural knowledge, credibility, and community accountability.

Yet this relational infrastructure remains precarious. These practices are rarely codified, systematically supported, adequately compensated, or protected within organizational structures. TIC therefore often operates as an unevenly distributed practice, dependent on individual labor rather than institutional commitment. Service continuity and quality become unstable when turnover disrupts relationships and removes tacit organizational knowledge. Agencies may experience reduced client engagement, breakdowns in care continuity, and increased compliance concerns after experienced staff depart (29). Variability in client satisfaction within the same organization reflects this same dynamic: outcomes are shaped less by standardized systems than by the relational capacities and discretionary practices of individual providers (29–31).

The paradox is that refugee-serving systems depend on a relational infrastructure they often fail to recognize or sustain. TIC is already present, but frequently carried by workers whose labor remains under-resourced and institutionally invisible. A structurally-informed approach should therefore move beyond asking whether providers are trauma-informed and instead ask whether organizations are designed to recognize, resource, and protect the relational labor through which TIC is actually delivered.

## 4 Toward structurally embedded trauma-informed systems

The limitations of TIC in refugee-serving systems point to a deeper conceptual issue: the unit of intervention remains the individual, while the conditions that produce and sustain trauma are collective and structural. Reframing TIC therefore requires a shift in the unit of care itself. This shift begins with a broader understanding of safety. In refugee-serving systems, safety depends not only on respectful clinical or service encounters, but also on the conditions that make daily life predictable and dignified: legal security, stable housing, income access, health coverage, transportation, family unity, and social belonging (32–34). These conditions constitute collective safety because they are produced socially and institutionally, not only interpersonally.

Structurally embedded TIC therefore requires alignment across policy environments, community infrastructure, organizational practice, and relational care. At the policy level, this means reducing administrative friction and legal precarity as core conditions of health and recovery, including prolonged asylum processes, health coverage interruptions, repeated eligibility verification, and fragmented benefit systems. Administrative redesign is not peripheral to TIC; it is one-way systems that reduce the uncertainty and loss of control that sustain distress. At the community level, refugee-led organizations, CBOs, faith networks, diasporic associations, and mutual aid systems should be understood as co-producers of care rather than adjunct referral partners. These systems sustain well-being through shared language, cultural interpretation, practical support, and collective problem-solving (35, 36). At the organizational level, TIC cannot rely on training as the primary implementation strategy. Training may strengthen awareness, but its effects are limited when workloads are unmanageable, supervision is inconsistent, funding is unstable, or performance metrics privilege volume and compliance over continuity and trust. Organizational implementation depends on leadership commitment, reflective supervision, protected time for relational work, and quality improvement processes that examine whether service procedures reduce or reproduce fear, confusion, and exclusion. At the relational level, structurally embedded TIC depends on the workforce through which trauma-informed practice is enacted in everyday encounters. Staff with lived experience of migration or displacement often serve as cultural brokers, trusted intermediaries, and interpreters of institutional expectations. These contributions should be recognized as professional expertise rather than informal personal labor through fair compensation, role clarity, stable career pathways, reflective supervision, and meaningful participation in organizational decision-making. System alignment occurs when these levels reinforce one another. Policy reforms reduce instability; community partnerships strengthen trust and legitimacy; organizational structures make relational work sustainable; and frontline relationships translate broader commitments into concrete experiences of care.

## 5 Conclusion

Advancing TIC for refugees and migrants requires a shift from individualized, clinically bounded approaches toward structurally aligned systems of care. Trauma is continually shaped by administrative, legal, and socioeconomic conditions that organize everyday life after migration. A meaningful trauma-informed system therefore extends beyond mitigating individual suffering to transforming the conditions that reproduce distress. Such a shift requires aligning policy, organizational practice, funding, and community infrastructure around collective safety. It also requires recognizing the relational labor already sustaining refugee-serving systems and protecting it through supervision, compensation, career pathways, and institutional accountability. Only then can TIC become more than compassionate practice within constrained systems: a framework for building systems in which refugees and migrants are met with dignity, stability, and the conditions necessary for healing.
